# Photonic Therapy in Periodontal Diseases an Overview with Appraisal of the Literature and Reasoned Treatment Recommendations

**DOI:** 10.3390/ijms20194741

**Published:** 2019-09-24

**Authors:** Marco Giannelli, Massimo Lasagni, Daniele Bani

**Affiliations:** 1 Odontostomatologic Laser Therapy Center, Via dell’ Olivuzzo 162, 50143 Florence, Italy; 2 Consultant Engineer, 50100 Florence, Italy; maslasa@tin.it; 3 Department of Experimental and Clinical Medicine, Research Unit of Histology & Embryology, University of Florence, viale G. Pieraccini 6, 50139 Florence, Italy

**Keywords:** periodontitis, dental laser, light-emitting diode, photoablative therapy, photodynamic therapy, photo-biomodulation

## Abstract

Recent reviews and meta-analyses of the literature over the past quarter-century have failed to provide enough evidence to prove or disprove the actual utility of photonic therapy in periodontitis, alone or adjunctive to conventional approaches. This apparent paradox has been explained by the many physical, molecular, biological, anatomical, and technical variables of photonic treatments, which can differ in light-emitting devices (laser or LED), wavelengths, irradiation power and modes, clinical objectives, follow-up times, disease grading, and assessment methods. This multi-faceted, controversial scenario has led practitioners to underestimate the actual potential of photonic therapy in periodontal diseases. In this critical appraisal of the literature, we have briefly summarized the main photonic therapies and instruments used in Periodontology, highlighting their main characteristics and limitations. Then, we have tried to identify and discuss the key methodological issues which can have an impact on the outcome of photonic therapies. Our main goal was to identify the best parameters, settings, and methodologies to perform effective periodontal photonic treatments and to extrapolate some recommendations for clinical use. Should these recommendations find a consensus among periodontologists and be adopted in future clinical studies, they will hopefully contribute to dissipate the present confusion and uncertainty on this complex matter.

## 1. Introduction

In a recent review dedicated to the most important advancements in periodontology over the past quarter-century, Professor C.M. Cobb underlines the fact that, in this period, the accumulated reports and clinical studies have failed to provide enough body of evidence to prove or disprove the actual utility of laser therapy in periodontitis [1]. As yet, the meta-analyses conducted on more than one hundred human clinical studies have not collected sufficient statistical evidence to suggest that integration of a laser, either as a monotherapy or as an adjunct to surgical and nonsurgical therapy in a periodontal treatment protocol will provide clinical outcomes superior to those achieved by traditional therapy based on scaling and root planing (SRP) [2]. It seems paradoxical that the many scientific data made available over time have not delineated a clear trend, not to mention broadly shared indications and protocols for laser therapy. In fact, the use of light for curative purposes, or ‘photonic therapy’, is intrinsically complex because of many physical, biological, anatomical, and technical variables which can have a profound influence on the final effect. The many diverse photonic treatments, exploiting different light-emitting devices (laser and LED), wavelengths, beam power, and irradiation modes, as well as the heterogeneous treatment protocols, exact biological rationale, follow-up times, disease grading, and assessment methods all contribute to make the literature on the clinical effects of photonic therapy in periodontal treatment controversial and difficult, if not impossible, to interpret [1]. Unfortunately, this leads dentists to underestimate the actual potential of this therapeutic approach in periodontal diseases. 

This article is not intended as a replica of the numerous excellent reviews and meta-analyses written by eminent periodontologists, to which we refer the reader who wishes to study this matter in greater depth [1,3,4,5,6,7,8,9,10]. Having been involved for several years in the study of photonic therapies in periodontics, we have felt the need for standardized, specific guidelines for the correct delivery of such therapies, particularly as an adjunct to conventional SRP, to provide non-invasive, patient-friendly, effective periodontal care [6]. In this critical appraisal of the literature available in the PubMed database (published in English), both reviews and primary clinical research, we have briefly summarized the main photonic therapies and instruments used in Periodontology, highlighting their main characteristics and limitations. Then, we have tried to identify and discuss the key methodological issues, emerged from our and others’ studies, which can have an impact on the outcome of the photonic therapies. Our goal is to elicit the attention of periontologists towards these points when planning a periodontal photonic treatment. Should these points become a shared base, they could be adopted as recommendations for future clinical studies, hopefully contributing to the disambiguation of the actual therapeutic value of the photonic approach. 

## 2. Photonic Therapy and Devices 

The premise for and basis of photonic therapy is that the light is used to improve the patients’ state of disease. For this reason, the light itself is currently viewed as the main subject while the devices used to administer it have been passing into the background. In fact, understanding of the physical properties and parameters of light is an obligatory step when setting up a photonic treatment and maximization of its therapeutic effects is chiefly if not exclusively related to the correct setting of key parameters such as: (i) Wavelength (λ), (ii) pulsed or continuous mode, (iii) intensity and dosage, (iv) fluence, (v) time, (vi) optical properties of the tissue itself, (vii) scattering of light within tissue, and (viii) absorption of the light by chromophores (hemoglobin, myoglobin, and melanin) [11]. Therefore, these parameters should always be taken into account and adjusted to the subjects, target tissues and therapeutic objectives. That said, the devices currently used for photonic therapy in periodontics are basically lasers and LED illuminators.

Lasers have been the first instruments developed for photonic therapy because of their unique properties of emitting a collimated, coherent light beam with monochromatic synchronous wavelength [12,13]. These characteristics allow medical lasers to be particularly versatile, since they can operate with specific wavelengths, high-energy or low-energy light beam, continuous, or pulsed wave mode, thus allowing a fine tuning of irradiation on the target tissues [3,13]. In particular, based on the energy level of irradiation, laser-based therapy can be distinguished between photosurgical/photoablative and low-energy therapy, also called low-level laser therapy (LLLT), which encompasses the phototherapic, photodynamic and photobiomodulating modalities. 

In recent years, for low-energy photonic therapy, non-coherent light sources such as light-emitting diodes (LED) have become increasingly common. The recent advancements in LED technology have consolidated their obvious advantages over lasers: negligible safety concerns, suitability for wearable devices and home use, possibility to irradiate large tissue areas at once and—last but not least—much lower costs of LED-based irradiation instruments [14].

## 3. Photoablative Therapy

This approach, requiring the use of lasers, exploits a high energy transfer to the target tissue to achieve its selective destruction, thus substituting the surgical scalpel: in addition, it also offers excellent hemostatic and bactericidal effects, leading to less inflammatory reaction and faster healing. Although high-energy lasers can also be used for soft and hard tissue photo-surgery, in the field of periodontology non-surgical therapeutic approaches are most common: Therefore, they will be the focus of this article. Energy absorption by the target varies depending on wavelength [15]: Thus, the lasers emitting in the far or intermediate infra-red spectrum, such as CO_2_ (λ = 10,600 nm) and Erbium family lasers, including Erbium-doped Yttrium-Aluminium garnet (Er:YAG; λ = 2940 nm) and Erbium-Chromium Yttrium Scandium Gallium garnet (Er:YSGG; λ = 2780 nm), are absorbed primarily by H_2_O (and secondarily by apatite) and operate chiefly through vaporization, whereas those emitting in the near infra-red spectrum, such as Neodymium-doped YAG (Nd:YAG; λ = 1064 nm) or diode (λ = 655–980 nm) lasers, are absorbed by tissue macromolecules and pigments and operate chiefly by coagulation/carbonization [3]. The different wavelengths also influence other parameters, such as light delivery by direct beam or optical fiber, penetration depth, light scattering, and related undesired thermal side effects, which render each laser type best suited for different, specific applications in the field of periodontology. Because of the narrow gap between therapeutic and unwanted side effects, the use of photoablative devices should be restricted to expert practitioners upon extensive training.

*Erbium family lasers* (λ = 2940–2790 nm). These lasers, whose photonic energy can be delivered either in contact or in non-contact mode, are mainly used to remove calculus, necrotic cementum, bacterial biofilm and endotoxins from the root surface and for alveolar bone surgery [16]. Due to its high absorption in water, Er:YAG laser induces sudden photo-thermal evaporation of the water contained into the mineralized tissues, leading to micro-explosions and ablation of a thin layer of the targeted tissues [17]. High water absorption is inversely proportional to reflection, scattering, and transmission of the infra-red radiation, which accounts for minimal energy spreading and thermal side effects in the surrounding tissues, not exceeding a range of 50 μm [18]. Pre-clinical and clinical studies have shown that Er:YAG laser can remove calculus with similar efficacy as SRP [19,20,21,22,23], albeit at the expense of an increased loss of cementum and dentin [24]. To limit undesired removal of root tissues, a combination of pulsed emission at frequency >10 Hz and low energy (40–100 mJ/pulse, energy density 10–20 J/cm^2^) is recommended [3,25]. Several studies emphasize the relationship between increasing power and energy density and increased removal of root surface tissue [24,26,27]. Water spray cooling is also recommended to further reduce undesired thermal effects, albeit with the caveat that adequate water cooling in deeper periodontal pockets is probably negligible. In turn, water can enhance the laser effects as it results in a micro-structured root surface [17,20,28,29] which may favor periodontium–root re-attachment and improvement of clinical attachment level (CAL) in the long term [22,30,31]. Of note, the clinical studies with sufficiently long follow-up periods have shown that Er:YAG laser, either in place of or in adjunct to manual or ultrasonic SRP, has similar efficacy to the conventional mechanical treatment in terms of standard periodontal markers probing pocket depth, bleeding on probing and CAL [30,32]. Conceivably, the long-term clinical efficacy of the Er:YAG laser is also related to its capability to eliminate periodontopathic bacteria and their endotoxins [33,34]: This property can positively influence the host–parasite balance and reduce destructive periodontal inflammation. In contrast to root photoablation, supra-gingival laser scaling on coronal enamel is contra-indicated, since complete calculus removal without affecting the underlying enamel is nearly impossible [3]. 

To enhance its precision, Er:YAG laser was coupled with a 655-nm diode laser to detect calculus by induced fluorescence, enabling effective removal of subgingival calculus while preserving integrity of the root surface [35]. This experiment demonstrates the usefulness of a diagnostic feedback system, a smart innovation that could be implemented in the next generation of photoablative lasers. 

On the other hand, Er:YAG laser is not the best choice for soft tissue surgery: despite the fact that abundance of tissue water allows easy and effective cutting of the gingiva and oral mucosa without substantial thermal damage, this laser, due to its micro-explosive mode of action, causes marked bleeding as well as splattering of cooling water and blood [3,36]. Being absorbed chiefly by water, its effects are not substantially influenced by tissue pigments such as hemoglobin and melanin, requiring no substantial adjustments of irradiation parameters related to local hyperemia or pigmentation [37]. 

Based on a selection of recent publications, in which the authors have reported the parameters and settings used to deliver an effective treatment in sufficient detail to allow anyone to repeat the treatment with any Er:YAG laser device [16,38,39,40,41], the suggested settings of photoablative Er:YAG laser for dental root debridement are summarized in Table 1.

*CO_2_ laser* (λ = 10,600 nm) is readily absorbed by water and is very effective for soft tissue surgery, having excellent hemostatic and bactericidal effects [3]. At variance with Er:YAG laser, however, it is also absorbed by the inorganic components of dental hard tissues and bone causing immediate over-heating and severe thermal damage [42], which deeply alters the root surface and hinders successive periodontal re-attachment [43]. Because of this feature, CO_2_ laser is contra-indicated for mineralized tissue photoablation. The only suitable mode of use in periodontics is in pulsed-wave, defocused beam mode to eliminate bacteria and their endotoxins in adjunct to conventional SRP [3].

The use of photoablative CO_2_ laser for soft tissue periodontal treatment is limited and the existing literature does not suffice to suggest specific treatment recommendations.

Nd:YAG laser (λ = 1064 nm), generally operating in pulsed wave mode, is poorly absorbed in water and chiefly absorbed by pigments, mainly melanin: this allows laser beam energy to penetrate deeply into tissues and cause photothermal coagulation. This property renders Nd:YAG laser particularly suited for soft tissue surgery, being able to achieve a sharp incision, 0.3–0.8 mm deep, with little or no pain, immediate hemostasis, and excellent post-surgical healing [44]. These properties have also been exploited to achieve selective photoablation of the gingival epithelium, which can be more rapidly, safely, and effectively removed than with any conventional surgical approach, with no bleeding, no need for anesthesia and no injury of the underlying mucosal lamina propria [45]. On the other hand, the same energy transfer features pose major limitations to its use on dental hard tissues: although Nd:YAG has been shown to be capable of removing subgingival calculus, even if less effectively than mechanical SRP, and of promoting satisfactory periodontal decontamination and healing [46], the effective irradiation power is very close to that inducing severe thermal damage to dental root and pulp tissues, narrowing the therapeutic window to output power settings between 1.5 to 3 W (60–150 mJ/pulse at 10–20 Hz) [3]. This issue likely accounts for the controversial reports and overall limited clinical evidence regarding the actual usefulness of photoablative Nd:YAG laser in periodontology. Its use is discouraged as a monotherapy for root curettage and should be restricted to removal of the infected epithelium and granulation tissue in adjunct to SRP [3,9,45]. It is noteworthy that Nd:YAG laser has been the object of accurate clinical studies whose results have allowed to draw a detailed periodontal protocol termed ‘laser-assisted new attachment procedure’ (LANAP) [47]. Standardization of this procedure has allowed to consistently achieve satisfactory gingival re-attachment and periodontal healing [48].

Based on a selection of recent publications in which the authors have reported the parameters and settings used to deliver an effective treatment in sufficient detail to allow anyone to repeat the treatment with any Nd:YAG laser device [49,50,51,52], the suggested settings of photoablative Nd:YAG laser for soft and hard tissue treatment are summarized in Table 2.

Diode lasers (λ = 630–980 nm), also emitting in the IR spectrum, are particularly versatile since they can operate in continuous or pulsed wave mode and, being delivered through optic fibers, in contact or non-contact mode. Laser diodes are usually capable of operating at high pulse rate (up to tens of kHz) and small pulse width (up to 1 µs). Recently, several semiconductor manufacturers have proposed new diodes functioning at >10 MHz and pulse width < 10 nsec. Their IR radiation is poorly absorbed in water but highly absorbed by pigments, particularly hemoglobin and melanin, resulting in good penetration into tissues, albeit lesser than Nd:YAG laser [53]. On the other hand, when used in contact mode, diode lasers show the phenomenon called ‘hot tip’: this consists of carbonization of organic debris at the tip of the optic fiber generating a dark capping which, under irradiation, attains high temperature and works similarly to electro-cauterization [53]. Peculiarly, the operating wavelengths of diode lasers do not substantially interact with mineralized tissues, allowing safe usage of these devices for periodontal soft tissue surgery and sulcular debridement, being particularly suited to achieve tissue ablation, hemostasis and sterilization even in close proximity to dental roots [4,9,36]. Similarly to Nd:YAG, diode lasers (λ = 810 nm) have been successfully used for selective photoablation of the gingival epithelium, with excellent hemostasis, no perceived pain and minimal injury of the underlying mucosal lamina propria [36,45,54,55]. Since the rate of heat generation by diode lasers is higher than Nd:YAG [56], operation under airflow cooling is required to prevent unwanted thermal injury, especially when this is used in contact mode [36]. Conversely, most studies agree that diode lasers are not suited for calculus removal and dental hard tissue treatment because they are ineffective at low irradiation energy while they cause undesirable alterations of the root surface at high irradiation energy [23].

Based on a selection of recent publications in which the authors have reported the parameters and settings used to deliver an effective treatment in sufficient detail to allow anyone to repeat the treatment with any diode laser device [36,55,57,58,59,60,61,62], the suggested settings of photoablative diode laser for soft tissue treatment are summarized in Table 3.

Other lasers, such as alexandrite crystal laser and excimer laser emitting in the UV spectrum (λ < 400 nm), have been investigated for possible use in periodontics because of their ability to be absorbed by pigments in dental calculus, which could be selectively ablated [63,64], but the evidence collected so far is too preliminary to draw any conclusion on their efficacy, while the use of high-energy UV radiation has raised safety concerns [3]. 

At present, no clinical protocols and settings are available for these lasers.

## 4. Low-Energy Photonic Therapy

In a modern meaning, the term ‘low level laser therapy (LLLT)’ to designate the curative effects of low power or defocused laser light should be replaced with ‘low-energy photonic therapy’. In fact, whether the light source is a laser, LED or other device, the biological and therapeutic effects depend on the specific wavelengths and irradiation modalities used (chiefly intensity, fluence rate, and treatment duration). Rather, based on the achieved effects, it makes sense to distinguish low-energy photonic therapy into (i) phototherapy, (ii) photodynamic therapy (PDT), and (iii) photobiomodulation therapy (PBMT). In the field of periodontology, low-energy photonic therapies are never stand-alone protocols but are rather intended as adjunctive treatments to conventional and laser-aided primary therapies 

Phototherapy indicates a direct biological action of specific wavelengths. In periodontics, this is chiefly exploited to achieve antimicrobial effects, based on the occurrence of target pigments in sensitive bacterial species, including several periodontal pathogens. The IR wavelengths of Nd:YAG and diode lasers, even when used in photoablative mode, can scatter in the surrounding tissues where they are lethally absorbed by black-pigmented gram negative anaerobes, including *Bacteroides*, *T. denticola,* and *P. gingivalis*: this phenomenon may contribute to the reported improvement of clinical outcome in patients receiving adjunctive laser treatments compared with those who only received conventional periodontal therapy (e.g., SRP) [65,66]. This finding, however, has been questioned by other studies showing that subgingival bacteria usually do not produce dark pigments and those doing so are a minority of the total bacterial populations in periodontitis patients [67,68]. Given the hundreds of bacterial species in the human periodontium, it is unfeasible to achieve a complete, enduring pocket sterilization by means of IR lasers [1].

More encouraging perspectives are offered by phototherapy in the violet-blue spectrum (λ 405–520 nm), which has shown excellent bactericidal effects against both Gram-positive and Gram-negative pathogens [69,70]. The mechanisms of these effects have been only partly elucidated: Among the possible modes of action, violet-blue light has been shown to excite bacterial porphyrins to endogenously produce bactericidal reactive oxygen species (ROS) [71]. Of note, in vitro studies have demonstrated that numerous periodontal pathogens, both in pure culture or in dental plaque, underwent significant growth reduction and death upon irradiation with blue light (LED, λ 455 nm) [72]. Interestingly, blue light (LED, λ 405 nm) at bactericidal dose has no effect on mammalian cells, which are protected from the ROS-dependent oxidative stress by potent endogenous anti-oxidant mechanisms [73]. This unique feature accounts for substantial efficacy and safety of blue light for oral disinfection. An additive beneficial effect of blue light (LED, λ 405 nm) consists in inactivation of lipopolysaccharide (LPS) [74], a Gram-negative endotoxin which plays a major role in maintenance of inflammation and periodontal tissue destruction in chronic periodontitis [75]. Obviously, as any antiseptic treatment in periodontology, blue light phototherapy needs to be repeated until a satisfactory clinical effect is achieved. 

**Figure 1:**
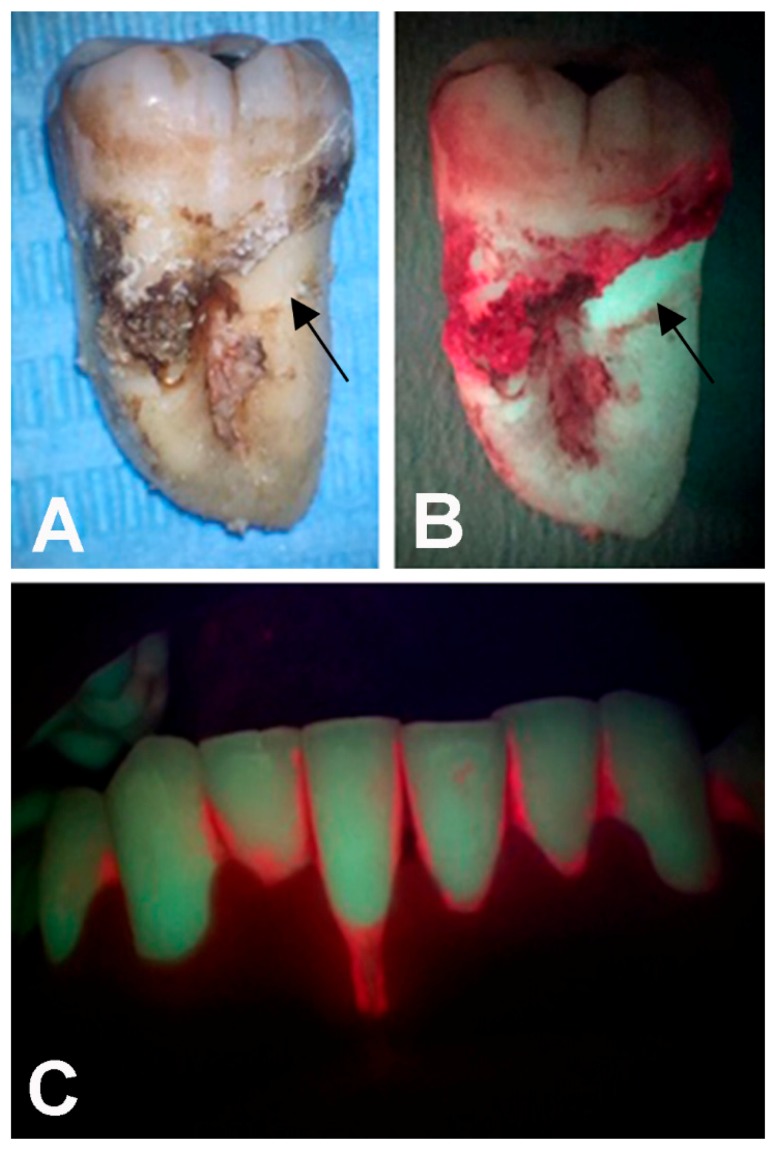
(**A**,**B**): Photoablation of dental calculus and plaque by Erbium-doped Yttrium-Aluminium garnet (Er:YAG) laser on an extracted root. Arrows point at the treated area. (**B**) Red fluorescence of the contaminated root surface revealed upon irradiation with λ 405 nm blue light. (**C**) The same photodiagnostic approach is used to detect full-mouth dental plaque.

Blue light also has additional photodiagnostic properties: It was found that dental plaque often exhibits red fluorescence when irradiated with blue light at λ 405 nm. This phenomenon is related to endogenous porphyrins produced by certain periodontal microorganisms emitting in the red spectral region when excited with light ranging from λ 400 to 420 nm [76]. Thus, irradiation with a blue light LED of the dental and periodontal tissues can allow an accurate detection of contaminated sites, useful to set up and monitor the antiseptic treatments (Figure 1).

The use of phototherapy with λ 405 nm LED for supportive periodontal treatment is limited yet. Based on the positive results of our previous study [55], an effective setting can be as follows: beam power, 1 W; power density, 1.05 W/cm^2^; total energy density (fluence), 63 J/cm^2^; non-contact irradiation mode (10 mm from the tissue; spot area at the target, 95 mm^2^).

Photodynamic therapy (PDT) is based on the photochemical properties of certain organic compounds such as cyclic tetrapyrroles, boron dipyrromethene (BODIPY), fullerenes and phenotiazinic dyes, the latter including toluidine blue O, methylene blue and its derivatives [76]. These compounds, generally termed ‘photosensitizers’, are absorbed by bacteria in an inert form and are subsequently photo-activated, i.e. excited by light at appropriate wavelength to their reactive triplet state [77,78]. There are two sequential photochemical pathways for the anti-bacterial effects of photosensitizer-based PDT: The first step (type II photochemistry) involves generation of singlet oxygen, which readily reacts to generate hydroxyl radicals (OH-) and, in cascade, ROS (type I photochemistry), which are considered the main effectors of bacterial photodamage and cytotoxicity [77,79]. ROS are toxic to microorganisms mainly through oxidative damage of plasma membranes and DNA [80]. Moreover, they have a short half-life (about 0.04 µs) and limited radius effect (0.20 µm) which render them particularly effective in the infected area, where the photosensitizers accumulate, with negligible undesired side effects on the tissues nearby [81]. Of interest, some bacteria, especially Gram-negative ones, can induce dimerization of phenotiazinic dyes thereby enhancing light absorption, photochemical reactions and overall bactericidal effects [82].

In the last decade, numerous reports have shown that antimicrobial PDT can be a valuable therapeutic approach in periodontal diseases, as it is able to eliminate bacteria [83], inactivate their endotoxin LPS [84,85], reduce pro-inflammatory cytokines [86] and endothelial adhesion molecules [45]. These biological effects synergize to reduce inflammation and periodontal tissue destruction. Accordingly, multiple sessions of PDT adjunctive to surgical treatment for chronic periodontitis have been shown to significantly improve the main periodontal clinical parameters in short- and long-term follow-up [36,54,55,86,87].

**Figure 2:**
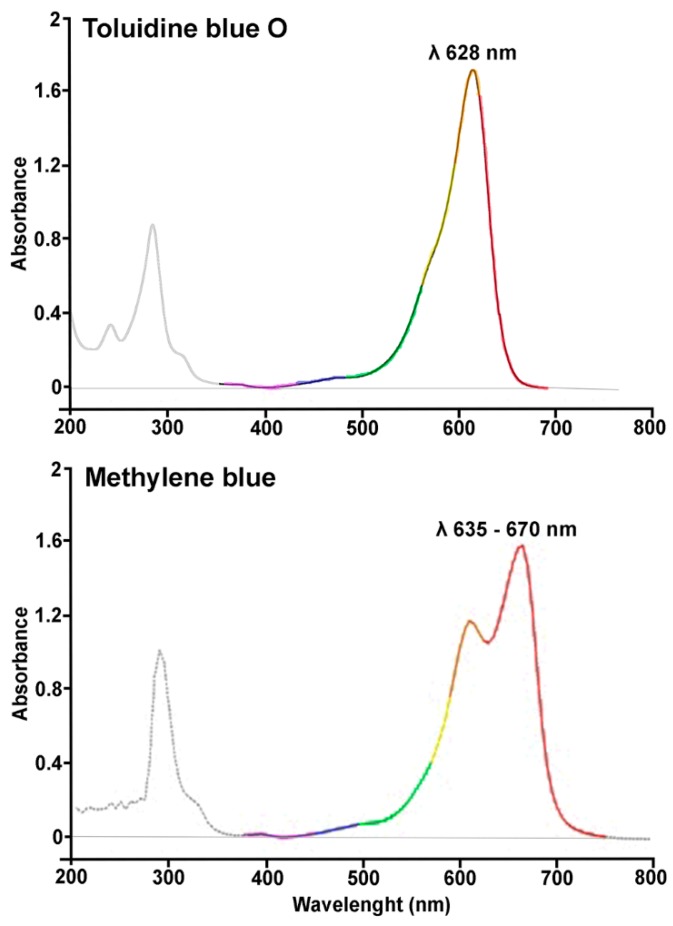
Absorption spectra of toluidine blue O and methylene blue, two phenotiazinic dyes most commonly used as photosensitizers in photodynamic therapy (PDT). Optimal photochemical reactive oxygen species (ROS) generation and anti-microbial effects are achieved with light wavelengths coincident with the absorption peaks.

A key issue in PDT is the choice of an appropriate excitation wavelength for the different photosensitizers. Each molecule is characterized by a definite spectrum of excitation wavelengths and the effective irradiation should fall within this spectrum, preferably close to the excitation peak. Thus, to mention the most commonly used photosensitizers, methylene blue shows two close absorption peaks at 635 and 670 nm, while toluidine blue shows a single peak at λ 628 nm [82,88]. The absorption spectra of these phenotiazinic dyes, reported in Figure 2, clearly show that both compounds can be photoactivated with light whose λ is encompassed between 550 and 700 nm. Therefore, the anti-bacterial effects reported in previous studies in which methylene blue or toluidine blue were used in combination with low-level IR diode lasers operating at λ 830 and 940 nm [89,90], which fall far beyond the absorption spectra of these photosensitizers, are not to be ascribed to PDT, but could rather be due to non-specific thermal effects. In this context, the careful choice of appropriate photosensitizers and the relevant irradiation wavelengths is mandatory when performing an effective PDT and would be desirable in order to gather objective and reliable clinical data on the actual efficacy of PDT in periodontology.

**Figure 3:**
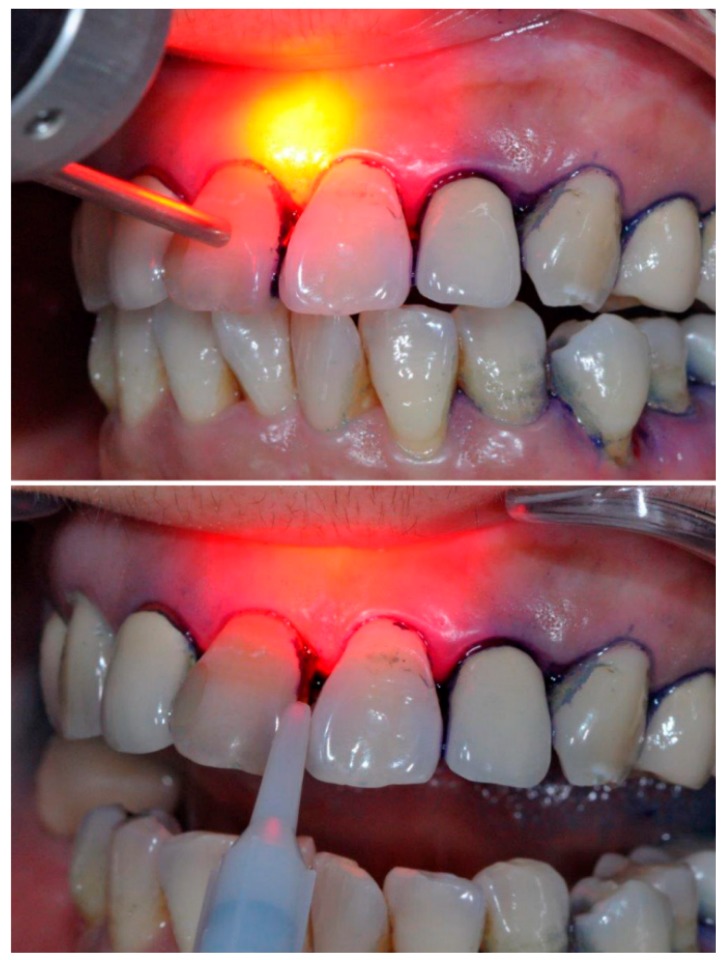
Photodynamic therapy (PDT) with toluidine blue O. Upper panel: Irradiation is correctly performed in non-contact mode using a λ 628 nm red light laser source and an adjustable-focus handpiece. Lower panel: Irradiation is performed in contact mode using the same λ 628 nm red light laser source and a Ø 800 μm optic fiber inserted into the periodontal pockets: this procedure should be avoided because it can cause harm and bleeding and reduce the efficacy of PDT.

Another key issue is related to the influence of the local conditions of the periodontal tissues to be treated. In fact, the efficacy of PDT is substantially reduced in the presence of bleeding and inflammation, since proteins in serum and exudate can compete in the tissue uptake of photosensitizers [91] and biological fluids can dilute them below the effective concentration. For the same reason, flushing out the photosensitizer solution before irradiation should be avoided, as should the insertion of the fiber tip in the periodontal pockets, as it can cause harm and bleeding. Rather, since red light can easily penetrate the tissues, photoactivation should be performed by transgingival irradiation in non-contact mode [92] (Figure 3). For the same reasons, in the presence of periodontal inflammation, more numerous PDT sessions and longer treatment times can be needed to achieve satisfactory results.

Based on a selection of recent publications in which the authors have reported the parameters and settings used to deliver an effective treatment in sufficient detail to allow anyone to repeat the treatment with any laser or LED device [55,85,93,94,95,96,97], the suggested protocol of PDT with λ 635 nm light source for supportive periodontal treatment is summarized in Table 4.

Photobiomodulation therapy (PBMT), broadly used for medical and veterinary purposes, usually exploits deeply penetrating IR wavelengths ranging between 600 and 950 nm to exert biostimulatory effects in the irradiated tissues, mainly consisting in increased cell proliferation and microvascular blood flow, which result in promotion of wound healing and bone regeneration and reduction of local inflammation, fibrosis and pain [5,98,99,100,101]. The cellular mechanisms underlying PBMT are many and only partially understood: Definitely, they are not dependent on mere thermal effects but seem to involve photochemical activation of cellular pigments, such as cytochromes in mitochondria, which enhance cell metabolism and availability of ATP and nitric oxide [5,102,103,104]. In the meantime, in vitro and in vivo studies have shown that irradiation with IR wavelengths used for PBMT abates the production of pro-inflammatory cytokines stimulated by bacterial LPS or surgical wounds [105,106], inhibits the transition of fibroblasts to pro-fibrotic myofibroblasts [107], promotes growth and differentiation of mesenchymal stem cells and osteoblasts [108,109], and modulates the levels of tissue plasminogen activator (tPA) and plasminogen activator inhibitor-1 (PAI-1) in periodontal tissues [110]. Consistently with the above notions, PBMT has been successfully used as co-adjuvant to conventional periodontal treatment to reduce periodontal inflammation, accelerate wound healing and gingival-root re-attachment, increase alveolar bone regeneration, and reduce post-operative pain [5]. However, the meta-analyses carried out on data from diverse reports have given inconclusive results, mainly because of dis-homogeneous clinical protocols [111]. This issue has prompted a re-appraisal of the right irradiation parameters for safe and effective PBMT [11]. As a final technical note, because of their many advantages over low-level lasers, LED devices are becoming preferable for PBMT purposes. Their simpler irradiation modalities should lead to more standardized clinical data and, hopefully, a clearer scenario on the benefits of PBMT in periodontics.

The protocols of PBMT with λ 635–810 nm laser or LED for supportive periodontal treatment have been summarized in a recent review [111].

## 5. Multi-Photonic Therapy

In recent years, evidence is emerging that satisfactory clinical results in periodontology can be obtained by a combination of multiple photonic treatments, in order to exploit the synergism between their different biological effects. In 2011 we first developed and applied in a pilot trial on patients with chronic periodontitis a multi-photonic approach adjunctive to SRP [36]. We started from the notion that periodontal tissue destruction occurs because of persistent inflammation related to unresolved infection by germs particularly adapted to the periodontal microenvironment, capable of penetrating and persisting into epithelial cells of the periodontal pockets and outer gingiva, thereby escaping host immunity and conventional anti-bacterial drugs [112,113] and predisposing the patients to re-infection soon after SRP [114], disease relapses, and chronicization [115,116]. Given this problem, we reasoned that a potential solution could consist in: (i) Laser photoablation of the infected epithelium, (ii) SRP for proper root cleansing, (iii) PDT with methylene blue in repeated sessions, to reinforce antisepsis and shift the parasite-host balance towards periodontal healing. This protocol, which we termed ‘PAPD’ for ‘photoablative-photodynamic’, met our expectations because it was shown to significantly improve the main periodontal clinical parameters in short- and long-term follow-up [36,54]. More recently, we have adopted a modified protocol, named iPAPD for ‘improved PAPD’, in which epithelial photoablation and SRP were followed by an intra-operative application of antiseptic phototherapy with a λ 405 nm LED (5 min), before the repeated PDT sessions. This improved protocol also gave satisfactory, statistically relevant clinical results in patients with severe periodontitis at 1-year follow-up [55]. 

The suggested iPAPD protocol for supportive periodontal treatment is summarized in Table 5.

## 6. Cellular and Molecular Effects of Photonic Treatments

The varying clinical effects of photonic treatments have their background in the diverse effects of light at the cellular level. Albeit the molecular aspects of the cell and tissue response to light mainly concern low-energy photonic irradiation, some of the collateral effects described upon photoablative laser treatments can be ascribed to the same mechanisms, since light can scatter and diffuse to the tissues nearby the area of impact of the laser beam. As briefly mentioned in the previous chapters, visible (λ 400–760 nm) and infra-red (λ > 760 nm) light can have clinically exploitable beneficial effects, which can be basically summary zed as: (i) Decreasing inflammation, (ii) analgesia, (iii) promoting tissue repair [117,118,119]. If overall irradiation power is low, e.g., <500 mW, no substantial heating of the tissues occurs and these effects can thus be considered light-specific, although they can substantially vary depending on the used wavelengths and irradiation parameters [11,80]. To exert cellular effects, absorption of photons of light radiation must occur. In fact, eukaryotic cells contain endogenous chromophores which behave as initial photoacceptor molecules: thence, the absorbed energy of photons is transferred to other molecules which play a role in cell function and metabolism [118]. The main chromophores and downstream molecular pathways activated by light can be resumed as follows [117].

(1) Cytochrome c oxidase (Cox): this is the final enzyme of the electron transport chain of oxidative phosphorylation in mitochondria which catalyzes the electron transfer from cytochrome c to molecular oxygen. Evidence has been offered that photons in the red/near infra-red λ can increase the availability of electrons, acting as up-regulators of mitochondrial membrane potential and hence ATP generation [120]. The exact molecular mechanism is yet unknown, although it could consist in photodissociation from the Fe^2+^ and Cu^2+^ centers of Cox of the inhibitor and rate-limiting factor nitric oxide (NO) [117]. Besides its obvious effects to support cell metabolism, increased ATP availability can be related to the light-induced rise in intracellular cAMP, which in turn regulates multiple cellular pathways involved in tissue repair and inflammation: For instance, cAMP inhibits TNF synthesis and, thus, down-regulates the inflammatory process [117,118]. Reduction of inflammation is also thought to be a main mechanism of the light-induced analgesia [117]. Increased mitochondrial activity also results in enhanced generation of superoxide anion and related reactive oxygen species (ROS), which in physiological concentrations can have stimulatory and signaling effects, promoting stem cell differentiation, increase in Ca^2^+ flow, mitogen-activated protein kinase (MAPK) activation and cell growth [119]. 

(2) Light-sensitive ion channels: these include for instance rhodopsin channels and transient receptor potential (TRP) channels, whose activation causes influx of cations, such as Ca^2+^, and changes in plasma membrane resting potential. Increased intracellular Ca^2+^ can modulate a number of cell responses, including for instance activation of cell proliferation and migration and release of tissue-trophic mediators from mast cells, which may concur to explain the tissue repair effects of light irradiation.

(3) Metal ion-containing enzymes, such as Cu–Zn superoxide dismutase and heme-containing peroxidase, involved in the regulation of intracellular redox potential and protection from inflammation-induced oxidative stress.

Besides these primary targets, other cellular pathways have been shown to be activated upon light irradiation, including expression of transcription factors, modulation of several cytokines and growth factors, and up-regulation of heat shock proteins, although the exact mechanisms remain to be fully elucidated [117]

It is known that if the incorrect parameters are applied, the photonic treatment is likely to be ineffective. In fact, too low or too high doses—in terms of fluence (J/cm^2^), irradiance (mW/cm^2^), irradiation time, or number of repetitions—can lead to no effects or even paradoxical inhibitory effects. This biphasic response, or ‘hormesis’, follows the Arndt–Schulz law, stating that weak stimuli slightly accelerate cellular activity, stronger stimuli raise it further until a peak is reached, whereas even stronger stimuli suppress it until a negative response is achieved [117]. This peculiar phenomenon can contribute to explain the above reported controversies about the clinical effects of photonic treatment in periodontal disease therapy.

## 7. Traps and Tips of Photonic Therapy Protocols

A careful analysis of the data from the literature has highlighted the profound differences between the several photonic therapies applied in periodontology, which in fact preclude to properly merge these data and identify the most suitable clinical protocols [1]. Focusing on these differences, however, enables us identify some key points which can be misleading if not properly taken into consideration when planning a photonic therapy protocol. Some of them are sketched out below.

Replacement or adjunctive to SRP. In periodontitis patients, debridement of the diseased root surface is usually performed by mechanical SRP using manual curettes or ultrasonic and air scalers. Although there is a general consensus that complete removal of bacteria and their toxins from the root surface and within the periodontal pockets cannot be achieved with conventional SRP, which provides the strongest rationale to photonic therapies, the greatest controversies deal with the actual advantages of replacing SRP with laser-aided debridement [1,3]. At present, only Er:YAG laser has shown the potential for effective calculus removal and root debridement capable of giving satisfactory clinical results in the long term [30,32]. However, use of this laser on dental hard tissues should be restricted to skilled operators, because it can easily result in increased loss of cementum and dentin and enamel damage [3,24]. Moreover, because of its narrow therapeutic window, the actual energy output of Er:YAG laser should be checked with a power meter at the fiber tip and adjusted before any treatment, in order to achieve the desired results. Because of these limitations and cautions, photonic therapy should be used in adjunct to conventional SRP rather than in its place for most periodontal applications [2].

Repeated irradiation, multiple sessions. Concerning low-energy photonic therapies, the previous clinical studies have indicated that, in many instances, their efficacy is modest and the differences with the control patients are often insignificant when they are delivered as a single treatment [85,86]. This is conceivable, taking into account that these photonic therapies are chiefly aimed at obtaining anti-bacterial effects. Repeated treatment sessions can result in progressive reduction of periodontal pathogens and their pro-inflammatory by-products, thus switching the host–parasite balance in favor of the former, eventually promoting periodontal healing [1,5]. On the above grounds, two key questions arise: how frequent and how many sessions. Frequency should be adjusted to prevent substantial bacterial re-growth between the sessions. Ideally, daily sessions are preferable, but patients may be annoyed by this: A possible solution would consist in portable photonic devices for self-administration of PT or PDT to patients at home, under medical prescription and control. Duration of the treatment should also be adjusted to each patient until satisfactory periodontal healing. In this context, a substantial body of evidence has indicated that the severity and clinical course of periodontal disease closely correlate with the local amounts and viability of the most aggressive periodontal bacteria, such as spirochetes [36,121], as well as of polymorphonuclear leukocytes (PMN) and erythrocytes which are reliable severity markers for periodontal inflammation and bleeding [36,54,55,122,123]. These parameters can be easily detected and quantified by cytological assay on smears of exfoliative material from the diseased periodontium [36,122,124] and assumed as indicators of periodontal healing to determine when PT or PDT can be safely discontinued [36,54,55]. Another feasible approach for evaluating periodontal healing could be to monitor inflammation biomarkers in the gingival crevicular fluid [125].

**Figure 4:**
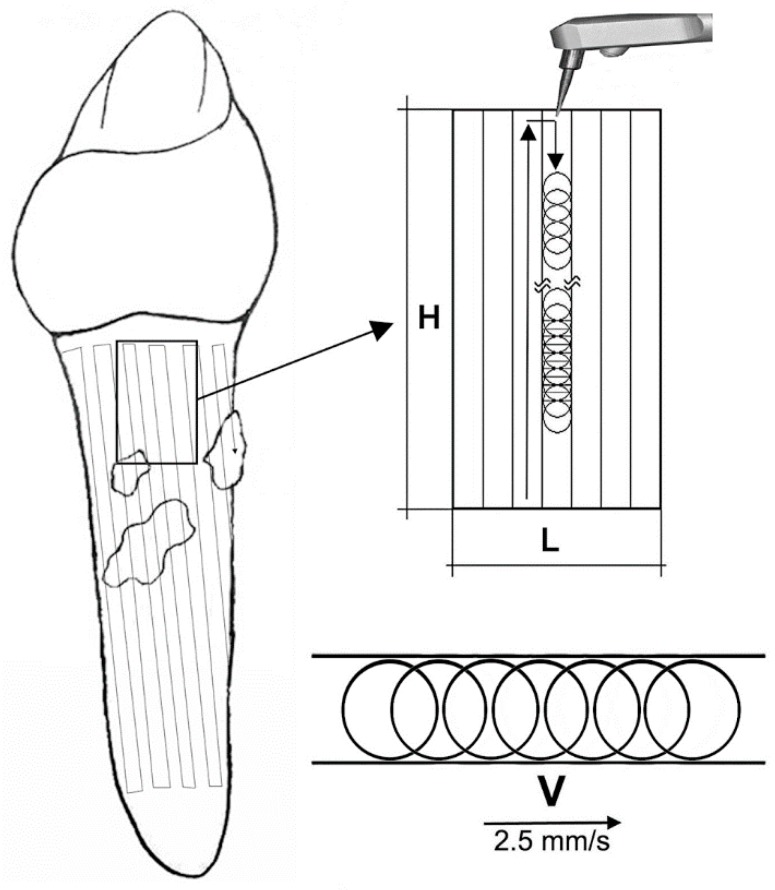
Graphic representation of the treated root surface during a photoablative treatment. Correct photoablation results from a succession of adjacent, non-overlapped lines covering the whole surface area under treatment. Accordingly, the total time of the treatment Tt is given by the following formula: $\mathrm{Tt}=N\;\times \;\mathrm{Ts}=N\;\times \frac{1}{S}$ where S is the constant speed of the laser spot, N is the number of lines, and Ts is the time required to treat a line.

Photoablative laser irradiation: Speed vs. time. Safe and effective photoablation depends on proper laser-tissue interaction. As discussed in the previous chapters, the physical characteristics of the different lasers and their irradiation settings and modalities need to be chosen and optimized based on the target tissue and the desired effects. A crucial point, often underestimated in many previous studies, is the movement of the laser beam, usually borne by an optic fiber, on the targeted tissue surface. Adopting speed as a main, objective parameter for target irradiation, photoablation results from a succession of adjacent lines covering the whole surface under treatment (Figure 4).

In a typical photoablative treatment, the fiber tip is moved to cover the total surface to be treated by drawing a sequence of contiguous stripes on the surface, as sketched in Figure 4, where v (considered as a mean value) is the tip speed. Surface shape is unimportant, while the relevant parameters are: area (S), number of stripes (N), and linear distance drawn by the tip (L).

**Figure 5:**
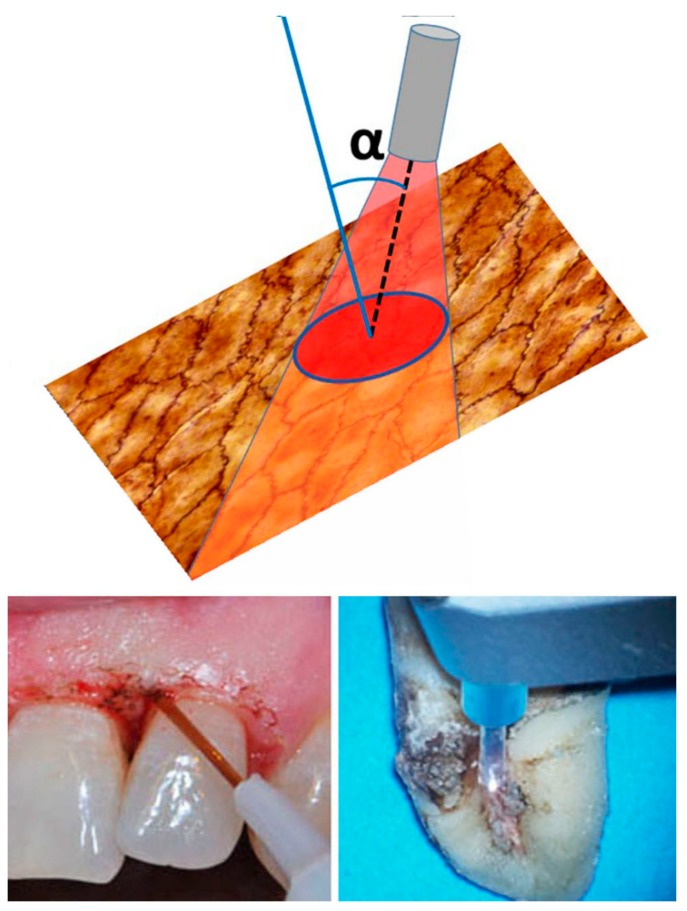
The diameter of the optic fiber, its angle (α) and distance from the target can deeply influence the actual irradiation energy delivered to the tissue. If the fiber is angled at 45°, the area of the irradiation spot increases and the overall energy is scattered proportionally. The below panels are representative of proper optic fiber angulation during diode laser photoablation of the gingival epithelium (left) and Er:YAG photoablation of calculus from a dental root (right).

In case of a rectangular surface and round beam cross-section, as shown in Figure 4, the following formula can be applied:

N = h/h_b_ (1)

where h is the height of the surface to treat and h_b_ is the length of the laser beam cross-section orthogonal to the movement line. Usually, in both contact and non-contact modes, the tip is not held perpendicularly but inclined by an angle α, as represented in Figure 5: in the upper panel, the rectangle represents the target tissue to be treated, the cone identifies the laser beam emitted from the tip, while the red ellipse represents the laser beam cross-section at the target surface (a circle section applicator is drawn, but similar considerations hold true for different types of applicators). The lower panels show the clinical implementation of photoablation with a diode laser inclined towards the gingival mucosa (left) and an Er:YAG laser inclined towards a dental root (right).

The surface interested by the laser beam on the tissue is larger than the tip section, thus the fluence at tissue will be lower than that at tip level. In particular, it can be calculated that beam cross-section at target level (*S_target_*) referring to the tip surface (*S_tip_*) and inclination angle (α):

(2) $$S_{target}=\frac{S_{tip}}{\cos\propto }$$

thus, fluence at target level (*F_target_*) can be calculated as:

(3) $$F_{target}=\;\frac{E_{p}}{S_{target}}=\;\frac{E_{p}}{S_{tip}}\cos\propto =\;F_{tip}\cos\propto$$

where *E_p_* is the laser beam (pulse) energy.

It is to remember that cos ranges from 0 (tip parallel to the surface, = 90°) to 1 (tip orthogonal o the surface, = 0°): in practice, with inclinations of 15°–20°, cos spans between 0.966–0.937, corresponding to a 3.4%–6% reduction. At 30º inclination, the reduction is 86.7%, while at 45° it becomes 70.7%.

Speed is the key parameter to be considered: for most purposes, 2.5 ± 0.5 mm/s is adequate to achieve effective photoablation while avoiding thermal damage of the target [126]. Operators can practice the correct speed on millimeter paper or other substrates, e.g., dental wax sheets, before implementing the treatments. Formulas to calculate the energy delivered to the target tissue as a function of speed have also been reported previously [126]. Care must be taken to not overlap the photoablation lines in order to avoid dual irradiation and possible tissue injury. Thus, the parameter ‘irradiation time’, often reported in previous studies to describe a photoablative procedure, becomes meaningless as it varies from case to case depending on the surface areas to be treated: In view of a better standardization of the photonic clinical protocols, its use should be discontinued.

The actual irradiation energy at the target. Another crucial point, which may have contributed to confound the results of the previous clinical studies, relates to the irradiation energy delivered to the target tissue, a key parameter for the efficacy of photonic therapies. In fact, especially for photoablative lasers, the actual energy of the light beam at the tip of the handpiece may differ substantially from the setting at the instrument’s display. Even when using new, high-quality optical fibers, energy at the laser source is reduced by about 30% during transmission through the fiber, not to mention the progressive use-related abatement of energy transmittance due to deterioration of the fiber material [38]. Because of these issues, before any photonic treatment, irradiation energy should be checked at the output from the terminal handpiece by a calibrated optical power meter, a low-cost instrument that should be present in the standard outfit of any photo-therapist, especially when administering high-energy lasers treatments. In fact, the effectiveness of a laser device is mainly affected by three key elements: (1) The laser source, where laser radiation is generated, (2) the delivery system, which transmits laser radiation to the applicator, (3) the tip of the applicator, which supplies laser radiation to the target. Faults, degradations or aging in any of them can result in improper treatments. Usually, the system control of a modern device is able to check the laser source and compensate some variations, usually the delivery system but not the tip. Verification of laser emission at the tip enables the operator to exclude malfunctions and assure the right instrument’s performance. Of note, the current regulations concerning medical laser systems establish that laser emission is correct if it is in the range of ±20% of the nominal set value. In normal use, this tolerance has little effect on the treatment. However, in research and experimental field, where the results of treatments are evaluated to define the right protocols, such differences are significant. Two different devices, even from the same manufacturer, with the same settings can yield completely different effects if their emissions are at the two sides of the acceptance range (they actually differ by 40%) and no measure of the exact emitted power is taken. In conclusion, it should be recommended that any new clinical studies report the exact values of irradiation energy at the target expressed as J/cm^2^. Moreover, as underlined in the previous chapter, speed of the fiber tip should be reported as well.

Other important parameters capable of deeply influencing the actual irradiation energy are the diameter (Ø) of the optic fiber, its angle, and distance from the target surface. Assuming 0 as distance (contact mode), a laser equipped with a Ø 300 μm fiber concentrates its energy on a spot roughly corresponding to the fiber cross-section area, i.e. 70,650 μm^2^; if this fiber is replaced with a Ø 600 μm fiber, the same energy is scattered on a 4-fold larger area, (282,600 μm^2^). Similarly, if the fiber is held perpendicular to the target surface, the irradiation spot roughly corresponds to the fiber cross-section area; if the fiber is angled at 45°, the area of the irradiation spot increases and the overall energy is scattered proportionally (Figure 5). Similar effect of light spot widening also occurs when the distance between the fiber tip and the target surface increases, due to the refractive index of interposed air.

Continuous wave, pulsed wave. Diode lasers are increasing their share in the market of dental lasers because of their favorable cost-performance ratio and versatility of use. In fact, these lasers can be set to emit a continuous or pulsed light beam [3]. The continuous wave mode enables the operator to easily estimate and control the energy delivered to the target tissue. The pulsed wave mode can be obtained in two different ways, gated pulse and free-running pulse: A gated pulse consists in chopping a continuous beam with a shutter operating at appropriate frequency, while a free-running pulse is generated by pulsations occurring in the laser tube. The gated pulse keeps the same maximum power of the continuous laser beam, while the free-running pulse allows to reach very high power peaks for very short times, with the advantage of high energy delivery to the target while minimizing undesired thermal side effects. There are no clear data helpful to define when continuous or pulsed wave modes should be preferred [11]: For the different photonic treatments, the choice of either modality should be checked towards the achieved results. In principle, in comparison with the continuous wave mode, the pulsed mode allows to increase the peak photon intensity, or irradiance (W/cm^2^), while maintaining the same overall dose, or fluence (J/cm^2^), and hence thermal effects.

## 8. Conclusions

Periodontologists are equally divided between supporters and skeptics about the usefulness of photonic therapy, the discriminating factor being basically dependent on individual positive or negative experience. Regretfully, as analyzed in the introduction, the excess of variables—not to mention some overt mistakes—among the diverse therapeutic protocols of the previous clinical studies have precluded to gain a sufficiently robust, statistically significant body of evidence in support to the photonic approach [1,2]. However, a careful, critical appraisal of the data from the literature has allowed us to identify several key points which, if they had been taken into proper consideration, could have made the difference between correct and erroneous photonic procedures. Two of these points appear particularly important: i) To be well aware of the makings and limitations of the different photonic devices and techniques and to use them for the right purposes; ii) to report in detail any methodological information needed to allow anyone to deliver a similar treatment with any laser or LED device. In this line of thought, compliance with these points should be adopted as benchmark to distinguish between correctly performed clinical studies to be included in future meta-analyses and wrongly designed studies reporting misleading data which should rather be excluded a priori. For instance, this criterion could be added to those in the AMSTAR checklist for methodological assessment of systematic reviews of clinical studies [127]. In prospective, the reasoned recommendations proposed here for proper settings of the different photonic instruments and procedures might desirably represent a starting base for further discussion to eventually arrive to a more standardized, consensual application of photonic periodontal therapy protocols in future clinical studies. 

A major limitation of our analysis is the relatively small number of the clinical studies which complied with our selected criteria for inclusion: Namely, those reporting the exact biological and clinical rationale and detailed information on the irradiation parameters and settings used to deliver an effective photonic treatment. Nonetheless, we hope that this analysis may contribute to dissipate the present confusion and uncertainty on this matter and, consequently, make more dental practitioners confident towards the usage of photonic treatments in periodontics. If this would happen, it could be foreseen that the demand for more versatile, user-friendly and less expensive light-emitting instruments specifically designed for oral use could steadily increase, similarly to what is happening in other branches of Medicine. For low-energy photonic treatments, a possible scenario could be the development of new protocols for supportive treatment of patients with chronic periodontitis based on portable LED instruments suitable for self-medication and home use, under medical supervision, capable to reduce the conventional pharmaceutical approaches to inflammation and infection.

## Figures and Tables

**Table 1:** Suggested parameters for photoablative Er:YAG laser.

**Source (reference No.)**	[16]	[38]	[39]	[40]	[41]	**Overall suggestions**	
**Laser emission settings**							
λ (nm)	2940	2940	2490	2490	2940	**2490**	
Wave emission mode (continuous/pulsed)	pulsed	pulsed	pulsed	pulsed	pulsed	**pulsed**	
Pulse energy (mJ)	160	100 (71) *	160 (114–136) *	160	40	**40-160**	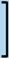
Frequency (Hz)	10	10	10	10	40	**40-10**	
Pulse width (ms)		0.25–0.5			0.1	**ND**	
Peak power (W)		400–200 (284–142) *			400	**≤400**	
Average power (W)	1.6	1.0 (0.71) *	1,6 (1.14–1.36) *	1.6	1.6	**≤1.6**	
**Applicator characteristics**							
Tip shape and size (mm)	chise l0.5 × 1.65 0.5 × 1.1	chisel 1.1 × 0.5	chisel 0.5 × 1.65 0.5 × 1.1	chisel	round 0.4	**round 0.5** **chisel 0.5–1.1**	
Beam divergence (degree)						**ND**	
Fluence at tip level (J/cm^2^)	19.39 29.91	18.18 (12.91) *	18.8–14.5		31.83	**≤50**	
**Laser application details**							
Application mode (contact/non-contact)	contact	contact	contact	contact	contact	**contact**	
Distance (mm)						**0**	
Laser spot size (mm^2^)						**ND**	
Beam inclination (degrees)	15–20	30	15–20	15–20		**15–20**	
Power density (W/cm^2^)						**ND**	
Energy density (J/cm^2^)		12.9				**ND**	
Treatment surface (cm^2^)						**ND**	
Tip movement speed (mm/s)						**ND**	
Total energy density (J/cm^2^)						**ND**	
**Treatment details**							
Treatment time (s) single (S)/multiple (M) dental roots	600 S 960 M	180–240		till smooth surface		**dependent on whole surface**	
No. of treatment	1	1	1	1	1	**1**	
Cooling system	water	water		water + air	water + air 28 mL/min	**water + air** **≥28 mL/min**	

Blue lines report the laser devices settings, yellow lines report data relevant to target irradiation, green lines report data relevant to the applied therapeutic procedure. Among the reported studies, there is a considerable variability in the mode, power, dose, number and frequency of treatment sessions, and not all the irradiation parameters and modes are consistently reported (blank boxes, not reported). Based on the results obtained in these studies, the right column extrapolates the suggested settings and parameters. Some of these are lacking (ND, not determined): the underlined ones are crucial and need to be specifically investigated by further studies. The values indicated by the square bracket are inversely related. * The values in parentheses correspond to those measured at the handpiece tip, while the preceding values are those indicated at the instrument’s display.

**Table 2:** Suggested parameters for photoablative Nd:YAG laser.

**Source (reference No.)**	[49]	[50]	[51]	[52]	**Overall suggestions**	
**Laser emission settings**						
λ (nm)	1064	1064	1064	1064	**1064**	
Wave emission mode (continuous/pulsed)	pulsed	pulsed	pulsed	pulsed	**pulsed**	
Pulse energy (mJ)	100	180–200	80	100	**80–200**	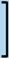
Frequency (Hz)	20	20	50	10	**50–20**	
Pulse width (ms)		0.1–0.15 0.55–0.65	0.35		**ND**	
Peak power (W)			240		**ND**	
Average power (W)	2	3.6–4.0	5	1	**≤5**	
**Applicator characteristics**						
Tip type, diameter (mm)/area (mm^2^)	optic fiber	optic fiber 0.36/0.10	optic fiber 0.6/0.28	optic fiber 0.2/0.003	**optic fiber** **0.3–0.6**	
Beam divergence (degree)					**ND**	
Fluence at tip level (J/cm^2^)		176.83–196.48	28.29	318.31	**≤200**	
Beam average power density (W/cm^2^)		3536–3930	1430	3183	**1400–4000**	
Beam peak power density (W/cm^2^)			85.800		**ND**	
**Laser application details**						
Application mode (contact/non-contact)	Contact	Contact	Contact	Contact	**Contact**	
Distance (mm)	0	0	0	0	**0**	
Laser spot size (mm^2^)					**ND**	
Beam inclination (degree)					**ND**	
Power density (W/cm^2^)			1430		**≤1400**	
Energy density (J/cm^2^)		12–17			**ND**	
Energy (J)			240–480 per tooth		**240–480**	
Treatment surface (cm^2^)					**ND**	
Tip movement speed (m/s)	slowly moved, circular		moved continuously	slowly moved sweeping	**ND**	
Total energy density (J/m^2^)					**ND**	
**Treatment details**						
Treatment time (s)			60–120 per tooth	120	**dependent on whole surface**	
No. of treatment	1	1	1	1	**1**	
Cooling system			water + air		**air**	

Blue lines report the laser devices settings, yellow lines report data relevant to target irradiation, green lines report data relevant to the applied therapeutic procedure. Among the reported studies, there is a considerable variability in the mode, power, dose, number and frequency of treatment sessions, and not all the irradiation parameters and modes are consistently reported (blank boxes, not reported). Based on the results obtained in these studies, the right column extrapolates the suggested settings and parameters. Some of these are lacking (ND, not determined): the underlined ones are crucial and need to be specifically investigated by further studies. The values indicated by the square bracket are inversely related.

**Table 3:** Suggested parameters for photoablative diode laser.

**Source (reference No.)**	[57]	[58]	[59]	[60]	[61]	[62]	[36,54,55]	**Overall suggestions**
**Laser emission settings**								
λ (nm)	810	980	810	980	940	810	810	**810–980**
Wave emission mode (continuous/pulsed)	continuous	pulsed	continuous	pulsed	pulsed	pulsed	continuous	Both*
Pulse width (ms)		10		25	20			**10–25**
Frequency (Hz)		30		13	25	50		**10–30**
Peak power (W)		1.5		2	1.5			**2.5**
Beam power (W) (average power if pulsed)	1	1.5	1.5	0.66	1.5	1	1	**1**
**Applicator characteristics**								
Tip type/diameter (mm)	optic fiber 0.6/0.28	optic fiber 0.4/	optic fiber 0.4/	optic fiber 0.3	optic fiber 0.3	optic fiber 0.3	optic fiber polyimide-coated silica 0.6	**0.4–0.6**
Beam diameter (mm)							0.28	**ND**
Beam divergence (degree)							16	**ND**
**Laser application details**								
Application mode (contact/non-contact) ‘hot tip’ inizialization (Y/N)	Contact Y	Contact Y	Contact N	Contact Y	Contact Y	Contact Y	Contact Y	**Contact** **Y**
Laser spot size (mm^2^)							0.28	**ND**
Beam inclination (degree)		20					15–20	**15–20**
Power density (W/cm^2^)			1193.7				353.4	**1193.7–353.4**
Total energy density (fluence) (J/cm^2^)					15		66.7	**66.7**
Laser tip movement, - time per area (s/cm^2^) - linear speed (mm/s)	vertical and horizontal lines	vertical and horizontal lines	vertical and horizontal lines	vertical and horizontal lines	vertical and horizontal lines 20 s/cm^2^	vertical and horizontal lines 2.5 mm/s	vertical and horizontal lines 2.5 mm/s	**vertical and horizontal lines 2.5 mm/s**
**Treatment details**								
Treatment time/tooth (s)	adjusted depending on the pocket’s surface area	60	20	20	20	20	adjusted depending on the pocket’s surface area	**adjusted depending on the pocket’s surface area**
No./Frequency of treatments	1	1	2	3	1	1	1	**1–3**
Cooling system	no	no	no	no	no	no	airflow	**airflow**
External power meter check	no	no	yes	no	no	no	yes	**yes**

Blue lines report the laser devices settings, yellow lines report data relevant to target irradiation, green lines report data relevant to the applied therapeutic procedure. Among the reported studies, there is a considerable variability in the mode, power, dose, number and frequency of treatment sessions, and not all the irradiation parameters and modes are consistently reported (blank boxes, not reported). Based on the results obtained in these studies, the right column extrapolates the suggested settings and parameters. Some of these are lacking (ND, not determined): the underlined ones are crucial and need to be specifically investigated by further studies. * Pulsed wave mode is preferable in case of gingival pigmentation.

**Table 4:** Suggested parameters for photodynamic therapy (PDT).

**Source (reference No.)**	[93]	[94]	[95]	[86]	[96]	[55]	[97]	**Overall suggestions**
**Laser emission settings**								
λ (nm)	670	670	660	670	660	635	660	**635/660**
Wave emission mode (continuous/pulsed)	continuous	continuous	continuous	continuous	continuous	continuous	continuous	**continuous**
**Applicator characteristics**								
Tip type/diameter (mm)	optic fiber	optic fiber	optic fiber 0.6	optic fiber 0.6	optic fiber 0.6	focalized zoom		**focalized zoom/** **optic fiber 0.6**
Fluence at tip level (J/cm^2^)	10–20		129	14.9	129	21		**21–129**
Power output (mW)	150		60	75	60	100	100	**60–100**
Power density (W/cm^2^)		0.075	21.4	1.2	21.4	0.35	0.25	**0.35–21.4**
**Laser application details**								
Application mode	transgingival	subgingival	subgingival	subgingival	subgingival	transgingival	transgingival	**transgingival**
Distance (mm)	7		0	0	0	30		**30**
spot diameter(mm)/area (mm^2^)	90					6/28,3		**ND**
Beam inclination (degree)						90		**90**
Energy density (J/cm^2^)				2.4		21	75	21–75
Treatment surface (cm^2^)						128	0.4	**ND**
**Photosensitizer**								
Metyhylene blue, MB Toluidine blue, TB	MB	MB	MB	MB	MB	TB	MB	**MB/TB**
Solvent	Periowave^TM^ solution	Helbo^TM^ solution	water	Helbo^TM^ solution	water	water	Na dodecyl-sulfate in water	**water**
Rinsing time pre-irradiation (min)		3	1	1	1	1		**1-3**
Concentration (mg/mL)	0.005	10 *	10 *	10 *	10 *	0.001	0.003	**0.001–0.003** **without rinsing**
Rinsing after application		yes	yes	yes	yes	no	no	**NO**
**Treatment details**								
Treatment time per tooth (s)	60	60	60	60	60	300	300	**adjusted depending on the area of each pocket**
Treatment repetitions	1	4	1	4	1	7–10	1	**4–10** adjusted depending on healing markers **
External power meter check	no	no	no	no	no	yes	no	**yes**

Blue lines report the laser devices settings, yellow lines report data relevant to target irradiation, green lines report data relevant to the applied therapeutic procedure. Among the reported studies, there is a considerable variability in the mode, power, dose, number and frequency of treatment sessions, and not all the irradiation parameters and modes are consistently reported (blank boxes, not reported). Based on the results obtained in these studies, the right column extrapolates the suggested settings and parameters. Some of these are lacking (ND, not determined). * Rinsing after application of the photosensitizer solution makes its actual concentration impossible to determine: in this instance, a large dye excess (up to 10 mg/mL) can be justified. ** Objective assessment of residual microbial/inflammatory markers, e.g., by cytosmear of pocket exfoliative samples, is recommended to check the effects of PDT and adjust its duration.

**Table 5:** Suggested parameters for multi-photonic (iPAPD) periodontal therapy [55].

	Photoablative Diode Laser	Phototherapy LED	PDT Diode Laser
**Laser emission settings**			
λ (nm)	810	405	635 Toluidine blue (1 μg/mL)
Wave emission mode	continuous	continuous	continuous
Beam power (W)	1	1	0.1
**Laser application details**			
Handpiece Diameter (mm)	Optic fiber 0.6	Adjustable focus lens	Crystal lightpipe 10
Application mode (contact/non-contact)	contact	non-contact	non-contact
Distance (mm)	0	10	30
Light spot size (mm^2^)	0.28	95	28.3
Power density (W/cm^2^)	353.4	1.05	0.35
Total energy density (fluence) (J/cm^2^)	66.7	63	21
Tip movement speed (mm/s)	2.5		
**Treatment details**			
No. of treatment	1	1	4–10 adjusted depending on healing markers *
Cooling system	airflow		

Blue lines report the laser devices settings, yellow lines report data relevant to target irradiation, green lines report data relevant to the applied therapeutic procedure. * Objective assessment of residual microbial contamination and inflammation, assumed as healing markers, was performed by cytosmear of pocket exfoliative samples, as described [36].
